# Impact of Individual Spinopelvic Anatomy on the Localization and Severity of Symptomatic Isthmic Spondylolisthesis

**DOI:** 10.1177/21925682231178206

**Published:** 2023-06-07

**Authors:** Kirsten Labbus, Justus Bürger, Jannis Löchel, Frederik Maximilian Schäfer, Michael Putzier, Robert Karl Zahn

**Affiliations:** 1 Center for Musculoskeletal Surgery (CMSC), Corporate Member of Freie Universität Berlin, Humboldt-Universität zu Berlin, and Berlin Institute of Health, 14903 Charité Universitätsmedizin Berlin, Berlin, Germany; 2 Institute for Radiology, Corporate Member of Freie Universität Berlin, Humboldt-Universität zu Berlin, and Berlin Institute of Health, 14903 Charité Universitätsmedizin Berlin, Berlin, Germany

**Keywords:** isthmic spondylolisthesis, spondylolytic spondylolisthesis, pelvic anatomy, spinopelvic alignment, pelvic incidence, sacral table angle

## Abstract

**Study Design:**

Retrospective analysis of prospectively collected data.

**Objectives:**

Isthmic spondylolisthesis (iSPL) occurs most commonly in L5/S1 and L4/5. This study investigates the association between spinopelvic anatomy and the pathogenesis of iSPL.

**Methods:**

Spinopelvic parameters as well as severity of slip grade were measured in sagittal spine radiographs of symptomatic patients with iSPL in segments L4/5 and L5/S1. Means were calculated and differences between both groups were analyzed. A correlation between the analyzed parameters and degree of slippage was performed.

**Results:**

We included 73 subjects in this study; 11 in L4/5 group and 62 in L5/S1 group. Pelvic anatomy significantly differed between L4/5 and L5/S1 iSPL (Pelvic Incidence (PI) 54.8° vs 66.3°, *P* value = .006; Pelvic Radius (PR) 124.4 mm vs 137.4 mm; *P* value = .005 and Sacral Table Angle (STA) 101.0° vs 92.2°, *P* value < .001). The relative degree of slippage was significantly higher in the L5/S1 group (L4/5 29.1% vs L5/S1 40.1%, *P* value .022). We also observed a significant correlation between pelvic anatomy and the severity of the slip in iSPL at the L5/S1 level.

**Conclusions:**

Pelvic parameters PI and STA play an important role concerning the level of occurrence and severity of iSPL. Spinopelvic anatomy determines the pathogenesis of iSPL.

## Background

Spondylolysis is a common cause of spondylolisthesis. Isthmic spondylolisthesis (iSPL) is defined as the translation of one vertebra in relation to the adjacent caudal vertebral body caused by an abnormality of the pars interarticularis. It occurs most commonly in level L5/S1 followed by L4/5.^1^ An iSPL in L4/5 seems to be more instable than in the segment below.^2,3^

While spondylolysis with or without spondylolisthesis is a common incidental finding in asymptomatic subjects with a prevalence between 4 and 8%, symptomatic patients present with chronic back pain, radicular leg pain or neurological deficits.^4^ Genetic predisposition, trauma, growth, morphological pelvic abnormalities, facet joint anatomy and biomechanical factors have all been implicated in the development of iSPL.^4,5^

Spondylolysis is a pathology exclusive to humans and has never been observed in newborns or non-ambulatory patients – this hints at the important role of biomechanical stress and the upright gait on development of iSPL.^6,7^

Pelvic anatomy determines spinopelvic alignment and subsequently the distribution of mechanical loading across the lumbar spine and the lumbosacral junction. Numerous studies show the influence of Pelvic incidence (PI) and individual spinopelvic interactions on the development of degenerative spinal diseases.^8-11^

In line with these findings, the influence of spinopelvic anatomy on the occurrence of L5/S1 Spondylolysis and iSPL has been established: iSPL is associated with greater PI, Sacral Slope (SS), Pelvic Tilt (PT) and Lumbar Lordosis (LL) compared to controls.^12,13^ There also seems to be an association between certain spinopelvic and sagittal alignment parameters of the spine and the occurrence of symptoms in spondylolysis and iSPL.^14-16^ It is suspected that some of these parameters correlate with the severity of symptoms and disability.^17^

A relatively large PI directly correlates to a large SS and physiological LL and associated with higher slip grades in iSPL, which further highlights the central role of pelvic geometry in the occurrence of iSPL.^18^

Even though it has been proven that sagittal alignment and spinopelvic anatomy play an important part in the development and clinical significance of spondylolysis and iSPL, little is known about the differences in spinopelvic alignment and anatomy between L5/S1 and L4/5 iSPL. Understanding the differences between iSPL in different levels of the lumbar spine might also be beneficial for the treatment of and surgical planning in symptomatic patients. The objective of this study is to investigate differences in spinopelvic parameters in isthmic spondylolisthesis in level L4/5 and level L5/S1. Secondary to observe differences in the correlation between spinopelvic parameters and the slip grade in L4/5 and L5/S1 iSPL.

## Patients and Methods

### Study Design

This is a retrospective analysis of prospectively collected data. In this study, we included patients with symptomatic iSPL who underwent lumbar fusion surgery at our institution between 03/2011 and 01/2019. Indications for surgery were iSPL in level L4/5 or L5/S1 and chronic back pain, pseudoradicular or radicular pain and/or neurological deficits that did not improve after at least 6 months of conservative treatment. Patients with a history of previous spinal surgery, tumour or trauma or insufficient radiographs (inadequate imaging of the hip joints in a preoperative lateral full spine or lumbar radiograph) were excluded from this study. We identified 85 patients with symptomatic iSPL in segment L4/5 or L5/S1 who underwent spinal fusion surgery at our institution between 03/2011 and 01/2019. Twelve patients were excluded due to inadequate imaging. The study was approved by the local ethics committee - due to the retrospective nature of this study, informed consent was not deemed necessary.

### Image Analysis

Preoperative lateral standing radiographs of either the lumbar or full spine including imaging of both hip joints were investigated for following spinopelvic parameters: Pelvic incidence (PI - angle between a line from the center of the bicoxofemoral axis to the middle of the sacral plateau and a line perpendicular to the sacral plateau), sacral slope (SS – angle between a line perpendicular to the sacral plateau and a horizontal line), pelvic tilt (PT - angle between a line from center of the bicoxofemoral axis to the middle of the sacral plateau and a vertical line), lumbar lordosis (LL), pelvic radius (PR - distance between the posterior superior corner of the sacrum plateau and the center of the bicoxofemoral axis), sacral table angle (STA - angle between sacral plateau and posterior wall of S1) and relative slip grade in percent. When full spine imaging was available C7 sagittal vertical axis (C7 SVA - distance between C7 vertical plumb line and the superior posterior corner of S1), T1 spinopelvic inclination (T1 Spi – angle between a line between the center of T1 and the center of the bicoxofemoral axis and a vertical line) and thoracic kyphosis (TK) were also measured. A spine surgeon and a radiologist performed the measurements. No significant differences between the measurements were found, so that the means were calculated and used for statistical analyses. Patients were split in 2 groups according to the affected segment: iSPL L4/5 and iSPL L5/S1. For radiographic measurement of the aforementioned parameters, clinical imaging tool Surgimap® (Nemaris Inc.; New York) was used.

### Statistical Analyses

All statistical analyses were performed using IBM SPSS statistical software version 25 (SPSS Inc., Chicago). For comparison of spinopelvic parameters between both groups, the two-sample *t* test (for parametric distribution) and Mann-Whitney U test (for nonparametric distribution) were used. The influence of gender was analysed by using Fisher´s exact test. Pearson correlation index was determined for metric parameters. Significance was considered at *P* value of < .05 for all tests.

## Results

In the study population (n = 73), 11 patients presented with iSPL in segment L4/5 (15.1%) and 62 patients had iSPL in segment L5/S1 (84.9%). Full spine radiographs were available in 62 patients (84.9%).

Genders were represented as follows: 33 women (5 in L4/5 (45.5%) and 28 in L5/S1 group (45.2%)) and 40 men (6 in L4/5 group (54.5%) and 34 in L5/S1 group (54.8%)). There was no significant difference between both groups in distribution of gender (*P* value = 1.00). Mean age was 53 years in L4/5 group and 47.5 years in L5/S1 group (*P* value = .293). In L4/5 group 4 patients had grade I slip and 7 patients had grade II slip (63.6%) according to Meyerding. Slip grade III and IV were not observed in L4/5 group. In L5/S1 group slip grade I was observed in 10 patients (16.1%), grade II in 41 patients (66.1%), grade III in 9 patients (14.5%) and grade IV or higher in 2 patients (3.2%). Demographics are shown in Table 1.

**Table 1.** Comparison Between Parameters of iSPL in L4/5 and L5/S1 Group.

Parameter	iSPL L4/5 (n = 11)	iSPL L5/S1 (n = 62)	*P* value
Sex [female/male]^a^	5/6	28/34	1.000
Age [years]	53.00 ± 11.75	47.50 ± 16.43	.293
PI [°]	54.76 ± 13.86	66.32 ± 12.29	**.006**
PR [mm]	124.38 ± 11.68	137.38 ± 13.81	**.005**
STA [°]	100.95 ± 5.58	92.23 ± 6.56	**<.001**
PT [°]	15.43 ± 9.52	20.82 ± 8.47	.102
SS [°]	39.33 ± 12.67	45.49 ± 8.63	.147
TK [°]	38.20 ± 13.01	35.05 ± 12.42	.508
LL [°]	-47.18 ± 34.30	-59.26 ± 10.84	.355
C7 SVA [mm]	48.78 ± 38.51	44.39 ± 39.35	.77
T1 Spi [°]	-.08 ± 4.20	-1.99 ± 4.34	.125
Slippage [%]	29.09 ± 8.41	40.05 ± 16.45	**.022**

^a^Values are given as the absolute number for each group.

All other values are given as the mean with standard deviation

**Figure 1.**
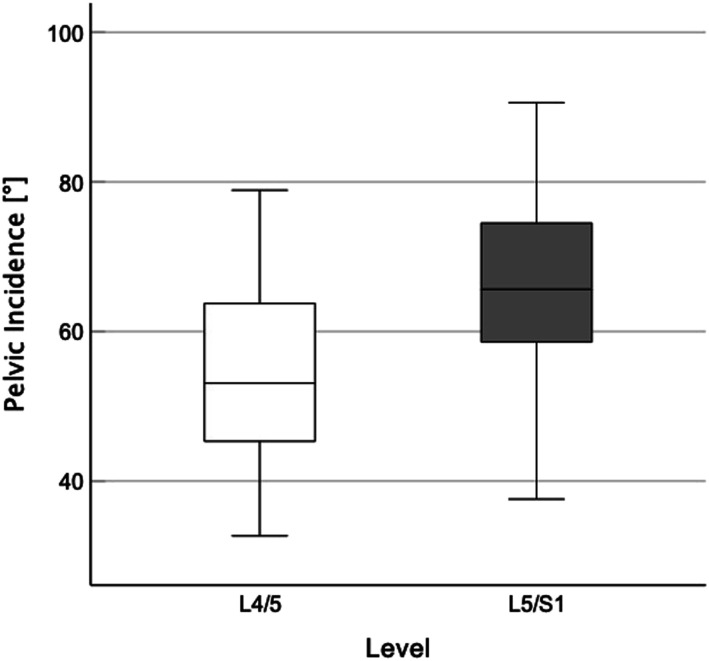
Box plot comparing pelvic incidence in Level L4/5 vs. L5/S1.

**Figure 2.**
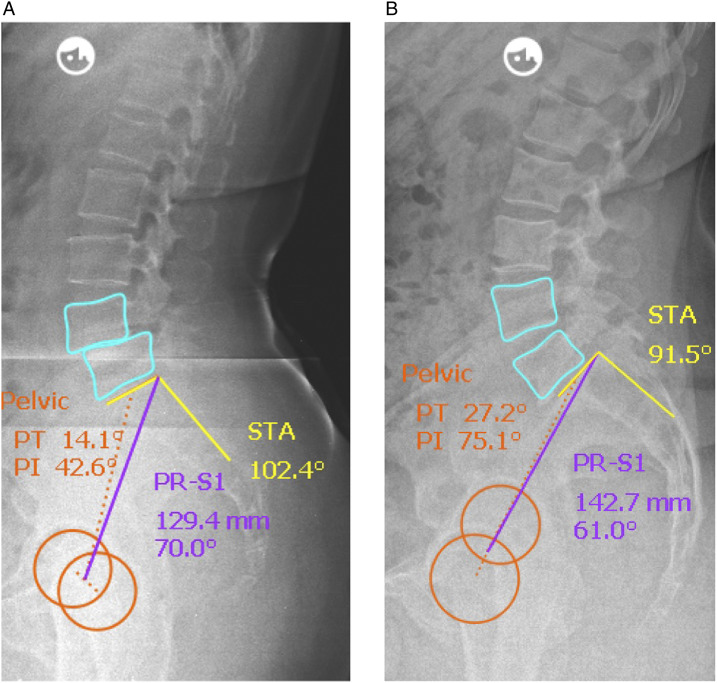
Radiographic examples of individual spinopelvic anatomy in sagittal radiographs for iSPL in L4/5 (a) and L5/S1 (b).

We observed significantly lower PI (54.8° vs 66.3°, *P* value = .006 – Figure 1) and PR (124.4 mm vs 137.4 mm; *P* value = .005) for L4/5 group than for L5/S1 group whereas STA was significantly higher in L4/5 group than in L5/S1 group (101.0° vs 92.2°, *P* value < .001). Figure 2a and 2b exemplary show comparative radiographs of subjects from each group.

For the following spinopelvic parameters no significant differences between L4/5 and L5/S1 groups were observed: PT (15.4° vs 20.8°, *P* value = .102), SS (39.3° vs 45.5°, *P* value = .147), TK (38.2° vs 35.1°, *P* value = .508), LL (−47.2° vs −59.3°, *P* value = .335), C7 SVA (48.8 mm vs 44.4 mm, *P* value = .770) and T1 Spi (−.1° vs −2.0°, *P* value = .125).

In our study population, we found a significantly lower severity of slippage in the L4/5 group compared to the L5/S1 group (29.1% vs 40.1%, *P* value .022). Positive correlation with the percental grade of slippage was found for PI (r = .385; *P* value = .002) in the L5/S1 group whereas no significant correlation was observed in the L4/5 group. Between STA and degree of slippage, we saw a negative correlation (r = −.548; *P* value < .001). In the L4/5 group, there was no significant correlation between the slippage and the STA.

## Discussion

This study provides relevant findings concerning the role of spinopelvic anatomy in the pathogenesis of symptomatic iSPL.

For the first time, it could be proven that spinopelvic anatomy differs significantly between the levels of iSPL and is associated with slip progression. Previous studies comparing spinopelvic anatomy in L4/5 and L5/S1 spondylolysis and iSPL without regard to severity did not report consistent differences between these 2 groups.^19^ Firstly, this can be attributed to the fact that they focused on a variety of mostly variable parameters like pelvic tilt, segmental or lumbar lordosis that – in the presence of spondylolisthesis – will most likely be changed as a compensatory mechanism and not represent the anatomy that promoted the occurrence of spondylolysis and spondylolisthesis. One study exclusively measured segmental lordosis and excluded all subjects with spondylolisthesis.^20^

While we also measured a number of local and global variable spinopelvic parameters, this study focuses on the (unchangeable) anatomic parameters Pelvic incidence, Sacral Table Angle and Pelvic radius. Pelvic Incidence and pelvic radius are well-established parameters that are often used to describe individual pelvic anatomy. We also included sacral table angle because of its role in development of iSPL.^21^ There is no fixed geometrical relationship between these 3 parameters. Interestingly enough, we found significant intergroup differences for all 3 parameters (PI, PR and STA). This fits the hypothesis that differences in individual spinopelvic anatomy cause a different distribution of biomechanical stress, which attributes to the occurrence of spondylolysis and spondylolisthesis in different segments of the lumbar spine. The theory is supported by finite element analyses of biomechanical forces in different sagittal alignment morphotypes that show a significant increase of forces on the lower lumbar segments with increasing pelvic incidence.^22^ We assume that the occurrence of iSPL in the segment L5/S1 is promoted by the increased mechanical forces caused by increased PI whereas other currently unknown factors cause the development of iSPL in segment L4/5. One possible factor that has been observed to favour development of L4/5 iSPL only and has not been assessed in this study is an increased body mass index at a younger age.^23^

Our study also shows that higher PI and lower STA correlate with higher grade of slippage in L5/S1 isthmic spondylolisthesis. These findings confirm results of other studies that have described a correlation between PI or STA and slip grade in isthmic spondylolisthesis L5/S1 and show a significant difference to L4/5.^13,19,24,25^ Although high correlation between 2 parameters does not imply causal relationship, it seems likely, that higher PI and lower STA lead to increased shear stress in the predisposed iSPL segment, which could explain a higher grade of shift between the vertebral bodies. The current literature hints at the fact that there is a difference between L4/5 and L5/S1 iSPL concerning the influence of spinopelvic anatomy on occurrence and severity of iSPL: Several studies have reported on the fact that a high Pelvic Incidence is associated with a higher degree of slippage in iSPL,^13,19^ but studies differentiating between the levels L4/5 and L5/S1 only reported this effect in L5/S1 iSPL.^18^ This shows that there might me a different mechanism promoting slip progression in iSPL L4/5 than L5/S1.

The incidence of L4/5 spondylolisthesis in our study population was 15.1%, which confirms the observation made in previous studies that iSPL most commonly appears in segment L5/S1.^3,23,26^ Our population included an almost equal number of men and women with iSPL which differs from other studies where a higher incidence of spondylolisthesis in male than in female subjects has been reported for the level L5/S1 (but not for L4/5).^4,23,27^ This might be related to the fact that our study population consists only of symptomatic subjects contrary to other studies investigating demographics of iSPL in asymptomatic subjects.

The study is limited by its retrospective design. Other factors that might determine the development of iSPL in different levels have not been evaluated in our actual study (eg ethnic background, level of physical activity, BMI). There might be some associations between other spinopelvic or sagittal alignment parameters and the development of clinically relevant iSPL, which have not been detected in our study. Secondly, our sample size, especially the number of patients in L4/5 group is low. This is caused by the fact that a clinically relevant iSPL in this level is less frequent than in L5/S1. However, due to the small sample size some statistical analyses might be artificial and type-II errors are possible.

In summary, individual sacral and pelvic anatomy differs between patients with iSPL L4/5 and L5/S1. According to PI as the determining factor for individual sagittal spinal alignment, differences in this spinopelvic parameter mean differences in sagittal lumbar alignment. Therefore, in clinical practice this fact should be considered for planning and execution and postoperative sagittal lumbar alignment should differ between both groups.

Further prospective studies (eg epidemiological studies) might help to confirm our findings of L4/5 iSPL characteristics. Investigating the specific spinopelvic parameters within a large cohort including symptomatic and asymptomatic subjects with iSPL would encourage the translation of our anatomical findings to a clinical setting.
